# Social determinants of violence against women in Panama: results from population-based cross-sectional studies and a femicide registry

**DOI:** 10.1093/inthealth/ihz116

**Published:** 2019-12-09

**Authors:** Ana Santamaría, Carmen Gerald, Fermina Chamorro, Víctor Herrera, Haydee Flores, Iris Sandoval, Beatriz Gómez, Eyra Harbar, Leoteau Liriola, Ruth G de León Richardson, Jorge Motta, Ilais Moreno Velásquez

**Affiliations:** 1 Gorgas Memorial Institute for Health Studies, Panama City, Panama; 2 Instituto Nacional de la Mujer, Panama City, Panama; 3 National Secretariat of Science, Technology and Innovation, Panama City, Panama

**Keywords:** intimate partner violence, Panama, social determinants of health, violence, women

## Abstract

**Background:**

We aimed to investigate the prevalence of violence against women (VAW) in Panama and its association with social determinants of health (SDH) and to estimate the femicide rates from 2014 to 2017.

**Methods:**

Data were derived from three cross-sectional population-based studies. Logistic regression models were used to estimate the association between SDH and VAW, expressed as odds ratios (ORs) and 95% confidence intervals (CIs). Age-standardized femicide rates were estimated using data from the Public Ministry.

**Results:**

Compared to the reference categories, women in the lowest quintile (Q) of income distribution (Q1: OR 4.0 [95% CI 1.4–11.7], Q2: OR 3.0 [95% CI 1.1–7.9]), divorced/separated women (OR 1.5 [95% CI 1.0–2.1]) and those in the age categories 25–33 y and 34–49 y (OR 1.9 [95% CI 1.2–3.1]) were more likely to have experienced violence in the past year. Indigenous ethnicity (OR 2.3 [95% CI 1.3–4.1]), age 15–19 y (OR 1.8 [95% CI 1.1–2.9]) and lowest education levels (very low: OR 4.7 [95% CI 1.4–15.5]; low: OR 4.5 [95% CI 1.4–14.6]) were associated with permissive attitudes towards violence. Indigenous (OR 2.7 [95% CI 1.3–6.1]), Afro-Panamanians (OR 3.1 [95% CI 1.3–7.6]) and education level (low: OR 2.5 [95% CI 1.2–4.9]; medium: OR 3.0 [95% CI 1.4–6.6]) were associated with physical/sexual intimate partner violence. Standardized adjusted femicide rates (×100 000) from 2015 to 2017 were 1.5, 0.9 and 0.8, respectively.

**Conclusions:**

Our findings highlight the importance of prevention programmes.

## Introduction

Violence against women (VAW) can occur throughout the lifespan and constitutes a violation of women’s human rights. VAW is defined as ‘any act of gender-based violence that results in, or is likely to result in, physical, sexual, or mental harm or suffering to the women, including threats of such acts, coercion or arbitrary deprivation of liberty, whether occurring in public or in private life’.^1^ One form of VAW is intimate partner violence (IPV), which refers to physical, sexual or psychological harm by a current or former partner or spouse.^2^ Globally, it is estimated that the lifetime prevalence of IPV among ever-partnered women is 30% and a total of 7.2% of women worldwide report ever having experienced non-partner sexual violence.^3^

VAW has long been considered a widespread yet largely under-reported public health problem with adverse health outcomes on physical, mental and reproductive health.^4–9^ Furthermore, intersections between VAW and violence against children have recently been documented.^10^ VAW remains widespread across Latin America,^11^ and consequently, if Sustainable Development Goal 5.2, which seeks to eliminate all forms of VAW, is to be achieved, it is essential to build evidence-based prevention programmes and policies.

Panama is located in Central America and has an estimated population of 4.2 million inhabitants. The country is geographically divided into 10 provinces and 5 *Comarcas*, which are areas populated by the native indigenous populations that represent 12% of the total population. Despite being classified as a high-income country, Panama has remained among the most unequal countries, with a Gini coefficient of 49.9 (2017).^12^ In 2008, the National Institute of Women (Instituto Nacional de la Mujer [INAMU]) was founded as a government entity that promotes gender equality, defends women’s rights and aims to prevent and eradicate VAW.^13^ In the year 2013, the legal framework started with a law that classifies femicide, defined as ‘the intentional murder of women because they are women’ as a crime.^14^ The establishment of a regulatory legal framework has been considered an integral step in the fight against VAW.^15^

Despite the legal advances, the system for collecting data regarding VAW in Panama is not yet unified at the public level. Therefore, surveys constitute an important source of information. In 2014–2015, a population-based study of women 15–49 y of age at the country level reported a lifetime prevalence of IPV of 34.0% and a 6.2% prevalence of sexual violence.^16^

Nevertheless, to date, the prevalence of VAW, risk indicators and regional differences in Panama have not been reported. Thus this study aims to investigate the prevalence of VAW in Panama and its association with social determinants of health (SDH) based on three population-based surveys. Furthermore, we aimed to estimate the rates of femicide from 2014 to 2017.

## Materials and methods

### Prevalence of violence and its association with SDH

We performed an analysis of data derived from three cross-sectional population-based studies: the Encuesta Nacional de Victimización y Percepción de Seguridad Ciudadana (ENVIP [National Survey of Victimization and Perception of Security]) study, the Multiple Indicator Cluster Survey (MICS) and the Encuesta Nacional de Salud Sexual y Reproductiva (ENASSER [National Survey of Sexual and Reproductive Health]).

The ENVIP study aimed to investigate criminal victimization, the experiences of people who have been exposed to a criminal event and the perception of the population towards public safety. The study base included citizens >18 y of age who resided in households in urban areas of Panama from June 2015 to May 2016. The data were collected during face-to-face interviews using a questionnaire. Among the 16 296 surveyed households, 13 802 interviews were completed (6135 men and 7667 women) (Supplementary Figure 1).

The MICS was performed with the aim of collecting international data about indicators of health and education in women, children and adolescents. The survey was conducted in 2012 and included households from urban, rural and indigenous areas. A detailed description of the study’s main results have been reported elsewhere.^17^ Briefly, women 15–49 y of age who resided in the selected household were invited to participate in the study and were asked to complete a questionnaire regarding information about access to media, sexual and reproductive health and domestic violence. In total, 9845 women were interviewed; of these, 9347 were included in our study (Supplementary Figure 2).

The ENASSER study was conducted in urban, rural and indigenous areas of Panama from 2014 to 2015.^16^ The aim of the study was to investigate indicators of sexual and reproductive health in women and men of reproductive age (15–49 y and 15–59 y, respectively).^16^ The data were collected during face-to-face interviews using a questionnaire. Of the 11 116 surveyed households, 5616 women participated in the study. Our study population included women whose privacy was ensured at the moment they began answering questions regarding VAW (n=4764) (Supplementary Figure 3).

### Outcome ascertainment

In the ENVIP study, the outcome analysed was past-year violence, defined as women’s self-reported exposure to physical or verbal violence by a partner or non-partner in the past year. If the study participant replied ‘yes’ to the following question: ‘Could you tell me if you were physically or verbally assaulted by someone known or unknown during the past 12 months’, she was categorized as having exposure to past-year violence.

In the MICS, the outcome of interest was permissive attitudes towards IPV. If the study participant replied ‘yes’ to one of the following questions, she was categorized as having permissive attitudes towards IPV: ‘It is fine for a woman to accept being hit by an intimate partner if 1) she goes out without telling him, 2) she neglects the children, 3) she argues with him, 4) she refuses to have sex with him, 5) she burns the food while cooking or 6) she dresses provocatively’.

In the ENASSER study, different outcomes were explored:

- Controlling behaviours by an intimate partner: defined as a woman answering ‘yes’ regarding any of the following situations: her partner gets mad or jealous if she talks to another man, he frequently accuses her of being unfaithful, he has stopped her from meeting her friends, he has tried to limit her contact with her family, he insists on knowing where she is all the time and he tries to stop her from going to work.
- Physical/sexual IPV: defined as a woman answering ‘yes’ to being exposed to any of the following situations by the current or previous partner: being pushed, shoved or having something thrown at her; being slapped; having her arm bent or her hair pulled; being hit with a fist or something else that could hurt; being kicked, dragged or beaten up; being choked or burnt on purpose; being threatened or attacked with a gun, knife or other weapon; being physically forced to have sexual intercourse when she did not want to; being forced to perform other sexual acts that she did not want to and/or being forced with threats to perform sexual acts that she did not want to.
- Physical violence during pregnancy: defined when a woman answered ‘yes’ to the following question: ‘During your pregnancy, did someone hit, slap or do something to physically hurt you?’
- Non-partner sexual violence: defined as a woman answering ‘yes’ to the following question: ‘At any time in your life, childhood or adulthood, has someone that was not your intimate partner forced you in some way to have sex or perform any other sexual act when you didn’t want to?’

### Definition of exposure variables

The SDH variables analysed in the present study include age, province, marital status, ethnicity, work status (in the ENVIP study only), monthly income and educational attainment. In the ENVIP study, the age categories were 18–24, 25–33, 34–49 and ≥50 y, whereas in the MICS and ENASSER studies the age categories were 15–19, 20–24, 25–33 and 34–49 y, as reported previously.^18^ The provinces were categorized into four groups according to a recently studied socio-economic index: indigenous areas (except the ENVIP study); Bocas del Toro and Darién; Chiriquí, Veraguas, Herrera, Los Santos, Colón and Coclé; and Panama province.^19^ Marital status was defined as being separated/divorced, married, single or widowed. Ethnicity was defined according to self-reported data as indigenous, Afro-Panamanian and other. Only in the ENVIP study did we explore the working status of the women interviewed. That variable was categorized as employed or unemployed, including retirees, ill-health pension, students, homemakers and women with a disabling condition that did not allow them to work (unable to work).

In the ENVIP study, monthly income (US$) was categorized into quintiles (Q): Q1, ≤$174; Q2, $175–$599; Q3, $600–$1499; Q4, $1500–$2999; Q5, ≥$3000. In the MICS and ENASSER studies, an index of household wealth has been calculated using principal component analysis and categorized into quintiles, as reported previously.^20^ While the province categories included average monthly income of the province as a constituent part of the index, the wealth index and the individual monthly income is a proxy of the individual and current socio-economic situation of the participant women.

Educational attainment was defined as very low (no school, pre-kindergarten and kindergarten), low (elementary school), medium (high school) and high (university, master’s and doctoral degrees).

### Femicide registry

To analyse the femicide rates, we used public data from the Statistical Centre of the Public Ministry. This entity has published a monthly report since 2014 consisting of the number of cases of femicide and the tentative cases of femicide by province and age.^21^ The report includes the number of domestic violence complaints filed by both sexes, by month and by province.

The present study was approved by the Ethical Review Committee of the Gorgas Memorial Institute for Health Studies, Panama.

### Statistical analysis

The survey data were weighted in order to represent the total female population in Panama (urban, rural and indigenous areas). Categorical variables are reported as proportions and continuous variables are reported as mean±standard deviation.

Unconditional logistic regression analysis was used to estimate odds ratios (ORs) and corresponding 95% confidence intervals (CIs) for the associations between SDH and the outcomes of interest. The reference categories were age >50 y, province of Panama, married marital status, ethnicity other than indigenous and Afro-Panamanian, employed, highest education level and Q5 of the monthly income/wealth index. Single adjustments for covariates were performed and combinations of covariates were adjusted (one by one) to evaluate their impact on the associations. We presented crude and adjusted ORs (adjusted by the potential confounding factors age, province, marital status, ethnicity, level of education and/or monthly income). Although socio-economic variables such as education and income might be correlated, they may also operate through different pathways. We examined the correlation between exposure variables (e.g. income and education) in each study and performed separate analyses that excluded highly correlated variables. In addition, all models were tested for multicollinearity.

The rates of femicide were analysed for the time frame 2014–2017, adjusted by age and standardized by the direct method according to the World Health Organization population estimates. The number of cases of femicide, tentative cases of femicide and reports of domestic violence were adjusted by the population density of each province according to the projections of the Panamanian Population Sampling Census in 2010.

All calculations were performed using Stata version 14 (StataCorp, College Station, TX, USA).

## Results

Tables 1 and 2 show the distribution of baseline characteristics of the study population of the ENVIP study, MICS and ENASSER.

**Table 1:** Baseline characteristics in the study participants of the ENVIP studies and MICS

ENVIP study	All (N=977 124)	Without prior violence* (N=895 734)	Past-year physical/verbal violence (N=49 944)	MICS	All (N=976 450)	Without permissive attitudes towards violence (N=913 805)	With permissive attitudes towards violence (N=62 645)
Weighted prevalence (%)		91.7	5.1	Weighted prevalence (%)		93.6	6.4
Age (years), %				Age (years), %			
18–24	14.6	14.3	13.9	15–19	16.1	15.5	24.7
25–33	17.7	17.3	23.1	20–24	15.9	15.9	15.2
34–49	30.5	29.9	37.1	25–33	22.5	22.8	18.9
>50	37.3	38.4	25.9	34–49	45.5	45.8	41.2
Province, %				Province, %			
Bocas del Toro and Darién	2.3	2.3	2.5	Bocas del Toro and Darién	4.8	4.4	9.8
Other provinces	28.6	29.1	22.3	Other provinces	34.8	34.8	35.4
Panama	69.1	68.5	75.2	Panama	54.8	56.0	36.3
				Indigenous	5.6	4.8	18.4
Marital status, %				Marital status, %			
Married/living together	56.8	57.6	50.9	Married/living together	59.1	59.3	56.4
Separated/divorced	19.0	18.2	22.5	Separated/divorced	12.8	12.7	15.5
Widowed	7.1	7.3	6.6	Widowed	1.0	0.9	1.3
Single	17.1	16.8	20.0	Single	27.0	27.1	26.9
Ethnicity, %				Ethnicity, %			
Indigenous	5.4	5.5	5.7	Indigenous	10.7	9.3	32.0
Afro-Panamanians	24.3	24.1	30.0	Afro-Panamanians	17.4	17.7	12.1
Other	70.3	70.5	64.3	Other	71.9	73.0	55.9
Working status, %				Working status. %			
Employed	45.7	45.1	53.2	Employed	–	–	–
Unemployed	54.3	54.9	46.8	Unemployed			
Level of education, %				Level of education^b^, %			
Very low	1.4	1.5	1.5	Very low	2.2	1.9	7.1
Low	15.9	16.3	11.6	Low	17.6	16.5	35.1
Medium	46.2	46.3	43.2	Medium	46.0	46.0	46.3
High	31.7	31.4	35.8	High	30.7	32.0	10.5
Very high	4.7	4.6	7.8	Very high	3.5	3.6	1.0
Monthly income^a^, %				Wealth index, %			
1st quintile	6.6	6.3	8.0	1st quintile	17.0	15.5	38.0
2nd quintile	33.8	33.8	35.5	2nd quintile	18.9	18.6	23.0
3rd quintile	41.1	41.4	35.4	3rd quintile	20.2	20.3	18.5
4th quintile	14.0	13.8	18.5	4th quintile	21.5	22.2	11.5
5th quintile	4.5	4.6	2.6	5th quintile	22.4	23.4	9.0

a 190 missing data: answered ‘I do not know’ or refused to answer monthly income.

b 7 observations excluded due to special education.

* Excludes those women who experienced violence prior to the past year (n=245).

**Table 2:** Baseline characteristics in the study participants of the ENASSER study.

	All women (N=1 145 101)	No controlling behaviours by the intimate partner (N=519 376)	Controlling behaviours by the intimate partner (N=343 726)^a^	No non- partner sexual violence (N=1 090 808)	Non- partner sexual violence (N=41 685)^b^	No physical violence during pregnancy^c^ (N=828 122)	Physical violence during pregnancy (N=21 243)	No physical/sexual IPV (N=788 748)	Physical/sexual IPV (N=74 689)^d^
Weighted prevalence, %		60.1	39.9	96.3	3.7	97.5	2.5	91.3	8.6
Age (years), %									
15–19	13.3	4.6	5.4	13.5	12.3	4.4	1.8	4.6	8.8
20–24	15.7	13.5	11.9	15.8	15.0	13.3	14.2	13.2	9.2
25–33	27.0	25.7	29.3	26.9	30.3	27.5	37.4	26.7	31.1
34–49	43.9	56.2	53.4	43.8	42.3	54.7	46.6	55.5	50.9
Province, %									
Bocas del Toro and Darién	4.0	3.5	4.8	3.9	7.3	4.0	11.0	3.6	7.9
Other provinces	28.3	26.9	30.9	28.2	36.8	28.9	49.4	28.3	30.0
Panama	63.1	64.7	59.2	63.2	52.6	61.7	33.2	63.1	55.9
Indigenous	4.6	4.9	5.1	4.6	3.4	5.3	6.3	4.9	6.2
Marital status, %									
Married/living together	63.2	83.5	84.4	62.8	70.1	80.4	67.0	85.5	69.9
Separated/divorced	11.7	15.9	15.0	11.6	10.4	12.7	29.2	13.9	29.6
Widowed	0.5	0.7	0.6	0.5	0	0.7	0.1	0.6	0.5
Single	24.6	–	–	25.1	19.5	6.2	3.7	–	–
Ethnicity, %									
Indigenous	10.5	10.6	10.9	9.8	19.4	10.8	19.8	10.0	18.6
Afro-descendant	30.4	25.6	28.4	30.7	22.5	27.2	20.4	25.2	42.6
Other	59.1	63.7	60.7	59.6	58.1	62.1	59.8	64.7	38.8
Educational attainment, %									
Very low	0.9	1.1	1.1	0.9	0.9	1.2	1.5	1.1	1.7
Low	10.9	12.2	14.9	10.5	19.9	13.2	31.4	13.0	16.1
Medium	56.6	50.6	55.0	56.9	44.2	54.2	51.0	50.7	69.6
High	31.6	36.1	29.0	31.7	34.9	31.4	16.1	35.2	12.5
Wealth index^a^, %									
Q5	5.9	6.0	7.3	5.9	3.9	7.0	8.5	6.3	8.9
Q4	7.7	8.2	10.1	7.5	11.4	8.8	18.4	8.6	12.9
Q3	7.7	7.4	7.9	7.7	11.4	7.8	15.5	7.5	8.7
Q2	34.0	32.9	37.8	33.9	30.2	36.0	32.7	33.4	50.4
Q1	44.6	45.5	36.9	45.0	43.1	40.5	24.9	44.2	19.1

a 1015 observations were excluded due to single status and 36 due to missing answer.

b 34 missing.

c 1085 excluded since prior pregnancy was denied.

d 1011 without ever having a partner excluded.

### Past-year physical or verbal violence

The proportion of women from the ENVIP study who experienced physical or verbal violence by a partner or non-partner in the past year was 5.1% (Table 1), whereas 3.2% (n=245) had experienced violence prior to the past year (Supplementary Figure 1). The correlation for income and education level was 0.46 and the variance inflation factor (VIF) was <10; therefore, the final model included both exposures. Compared with the reference categories, being divorced/separated (adjusted OR 1.5 [95% CI 1.0–2.1]) and being between 23 and 33 or 34 and 49 y of age were associated with violence in the past year (adjusted OR 1.9 [95% CI 1.2–3.1]). Similarly, women in the lowest quintiles of the income distribution were 3–4 times more likely to experience past-year (within the last 12 months) violence (adjusted Q1 OR 4.0 [95% CI 1.4–11.7], Q2 OR 3.0 [95% CI 1.1–7.9]). In contrast, an inverse association was observed between the province of Bocas del Toro and Darién (adjusted OR 0.6 [95% CI 0.5–0.8]) and past-year violence. Likewise, high, medium and low educational attainment showed an inverse association with our outcome of interest (Table 3).

**Table 3:** Association between SDH and violence against women in the MICS and ENVIP studies.

ENVIP	Past-year physical/verbal violence		MICS	Permissive attitudes towards IPV	
	OR (95% CI) (crude)	OR (95% CI) (adjusted^a^) (N=920 538)		OR (95% CI) (crude)	OR (95% CI) (adjusted^a^) (N=975 144)
Age category (years)			Age category (years)		
>50	1	1	34–49	1	1
18–24	1.4 (0.8–2.4)	1.4 (0.8–2.5)	15–19	1.7 (1.2–2.4)	1.8 (1.1–2.9)
25–33	2.0 (1.3–2.9)	1.9 (1.2–3.1)	20–24	101 (0.7–1.6)	1.3 (0.8–2.1)
34–49	1.8 (1.2–2.7)	1.9 (1.2–3.1)	25–33	0.8 (0.6–1.2)	0.9 (0.7–1.4)
Province			Province		
Panama	1	1	Panama	1	1
Bocas del Toro and Darién	0.7 (0.5–0.9)	0.6 (0.5–0.8)	Other provinces	1.6 (1.1–2.2)	1.4 (1.0–2.0)
Other provinces	1.0 (0.6–1.8)	0.8 (0.4–1.5)	Bocas del Toro and Darién	3.4 (2.3–4.9)	1.4 (0.8–2.5)
			Indigenous areas	5.9 (4.2–8.4)	1.5 (0.7–2.9)
Marital status			Marital status		
Married/living together	1	1	Married/living together	1	1
Separated/divorced	1.4 (0.9–1.9)	1.5 (1.0–2.1)	Separated/divorced	1.3 (0.9–1.8)	1.5 (1.0–2.2)
Widowed	1.0 (0.5–2.1)	1.5 (0.7–3.5)	Widowed	1.4 (0.5–3.9)	1.5 (0.5–4.3)
Single	1.3 (0.9 to 2.0)	1.3 (0.9 to 2.1)	Single	1.0 (0.7 to 1.4)	1.0 (0.6 to 1.6)
Ethnicity			Ethnicity		
Other	1	1	Other	1	1
Indigenous	1.1 (0.6–2.2)	1.1 (0.5–2.1)	Indigenous	4.5 (3.4–5.8)	2.3 (1.3–4.1)
Afro-Panamanian	1.4 (0.9–1.9)	1.3 (0.9–1.8)	Afro-Panamenian	0.9 (0.5–1.5)	1.0 (0.6–1.7)
Education level			Education level		
Very high	1	1	Very high	1	1
High	0.6 (0.4–1.1)	0.5 (0.3–0.9)	High	1.2 (0.4–4.1)	0.9 (0.3–3.3)
Medium	0.5 (0.3–0.9)	0.4 (0.2–0.7)	Medium	3.7 (1.2–11.7)	2.5 (0.8–7.9)
Low	0.4 (0.2–0.7)	0.3 (0.2–0.7)	Low	7.9 (2.5–24.8)	4.5 (1.4–14.6)
Very low	0.6 (0.2–1.9)	0.5 (0.1–1.9)	Very low	13.9 (4.4–44.1)	4.7 (1.4–15.5)
Monthly income (quintile)					
Q5	1	1			
Q4	2.4 (0.9–6.7)	2.9 (1.0–7.8)			
Q3	1.5 (0.6–4.1)	2.0 (0.8–5.3)			
Q2	1.9 (0.7–5.0)	3.0 (1.1–7.9)			
Q1	2.3 (0.8–6.5)	4.0 (1.4–11.7)			

^a^ MICS: point estimates in the adjusted model were adjusted by all variables except the wealth index.

### Permissive attitudes towards violence

The proportion of women in the MICS who disclosed having permissive attitudes towards violence was 6.4% (Table 1).

There was a positive moderate correlation between education level and the wealth index (0.61). When both variables were included in the models, the goodness-of-fit test was <0.05; therefore, the final model included only education level, because education has been linked to better-informed health-related decisions and quality of life.^22^ However, the point estimates did not change substantially when only the wealth index was included in the model.

Compared with the reference categories, being of indigenous ethnicity (OR 2.3 [95% CI 1.3–4.1]), being 15–19 y of age (OR 1.8 [95% CI 1.1–2.9]), being separated/divorced (OR 1.5 [95% CI 1.0–2.2]), living in other central provinces (OR 1.4 [95% CI 1.0–2.0]) and having a very low (OR 4.7 [95% CI 1.4–15.5]) or low education level (OR 4.5 [95% CI 1.4–14.6]) were associated with permissive attitudes towards violence (Table 3).

**Table 4:** Association between SDH and violence against women in the ENASSER study.

	Physical/sexual IPV		Physical violence during pregnancy	
	OR (95% CI) (crude)	OR (95% CI) (adjusted) (N=863 103)	OR (95% CI) (crude)	OR (95% CI) (adjusted) (N=849 365)
Age category (years)				
34–49	1	1	1	1
25–33	1.3 (0.4–3.5)	1.2 (0.5–2.8)	1.6 (0.8–3.3)	1.5 (0.7–2.9)
20–24	0.8 (0.2–2.3)	0.6 (0.2–1.7)	1.2 (0.4–3.7)	1.2 (0.4–3.5)
15–19	2.1 (0.6–7.6)	1.2 (0.3–4.8)	0.5 (0.1–1.7)	0.4 (0.1–1.4)
Provinces				
Panama	1	1	1	1
Other provinces	1.2 (0.5–2.9)	1.6 (0.9–2.8)	3.2 (1.7–7.7)	2.9 (1.2–7.0)
Bocas del Toro and Darién	2.4 (0.9–6.1)	1.8 (0.9–3.4)	5.1 (2.0–12.6)	3.1 (1.0–9.3)
Indigenous areas	1.4 (0.5–3.7)	0.6 (0.2–1.6)	2.2 (0.7–6.4)	1.2 (0.3–5.8)
Marital status				
Married/living together	1	1	1	1
Separated/divorced	2.6 (1.1–5.9)	3.1 (1.6–6.2)	2.8 (1.3–5.7)	3.0 (1.4–6.4)
Widowed	0.9 (0.2–4.0)	0.8 (0.2–3.6)	0.2 (0.03–2.0)	0.3 (0.03–1.8)
Single	–	–	0.7 (0.3–1.7)	0.7 (0.3–1.7)
Ethnicity				
Other	1	1	1	1
Indigenous	3.1 (1.9–5.0)	2.7 (1.3–6.1)	1.9 (0.9–4.0)	1.8 (0.5–6.9)
Afro-Panamanian	2.8 (0.9–9.2)	3.1 (1.3–7.6)	0.8 (0.3–2.2)	1.2 (0.4–4.0)
Education level				
High	1	1	1	1
Medium	3.8 (1.5–9.7)	3.0 (1.4–6.6)	1.8 (0.7–4.8)	1.8 (0.7–4.1)
Low	3.5 (1.8–6.7)	2.5 (1.2–4.9)	4.6 (1.7–12.6)	4.1 (1.2–14.2)
Very low	4.6 (1.8–11.2)	2.3 (0.8–6.3)	2.4 (0.5–11.3)	2.0 (0.3–11.4)
Wealth index (quintile)				
Q5	1	1	1	1
Q4	3.5 (1.2–10.5)	2.0 (0.7–5.7)	1.5 (0.5–4.1)	1.0 (0.3–3.1)
Q3	2.7 (1.4–5.1)	1.6 (0.7–3.2)	3.2 (1.2–8.8)	1.2 (0.5–3.3)
Q2	3.5 (1.8–6.8)	2.2 (0.8–5.3)	3.4 (1.3–8.8)	1.1 (0.4–3.4)
Q1	3.3 (1.7–6.2)	2.2 (0.7–6.4)	1.9 (0.8–5.1)	0.6 (0.1–2.1)

### IPV

The correlation for education level and wealth index was 0.46 and the VIF was <10; the final model included both exposures. In total, 3747 women reported having ever had a partner and were thus asked about partner violence (Supplementary Figure 3). The proportion of women in the ENASSER who disclosed experiencing controlling behaviours by their intimate partner was 39.9% (Table 2). No association was observed between exposure variables and controlling behaviours by the intimate partner (Supplementary Table 1).

A total of 8.6% of the women had experienced physical and sexual IPV by their current/last partner (Table 2). Divorced/separated women (OR 3.1 [95% CI 1.6–6.2]), women of indigenous (OR 2.7 [95% CI 1.3–6.1]) and Afro-Panamanian (OR 3.1 [95% CI 1.3–7.6]) ethnicities and those with low (OR 2.5 [95% CI 1.2–4.9]) and medium (OR 3.0 95% CI 1.4–6.6]) education levels were more likely to experience physical/sexual IPV (Table 4).

After the exclusion of women who denied prior pregnancy (n=1085), 2.5% of the women reported experiencing physical violence during pregnancy. Residence in Bocas del Toro or Darién (OR 3.1 [95% CI 1.0–9.3]) or other provinces (OR 2.9 [95% CI 1.2–7.0]), being separated/divorced (OR 3.0 [95% CI 1.4–6.4]) and having a low level of educational attainment (OR 4.1 [95% CI 1.2–14.2]) were associated with experiencing physical violence during pregnancy (Table 4).

Furthermore, 3.7% of the women were exposed to non-partner sexual violence. No association was observed between the exposure variables and non-partner sexual violence (Supplementary Table 1).

### Femicide registry

The standardized adjusted femicide rates (×100 000 women) for the years 2015–2017 were 1.5, 0.9 and 0.8, respectively. The most commonly recorded forms of aggression were stabbing (n=38 [36.2%]), shooting (n=26 [24.7%]) and asphyxiation (n=14 [13.3%]). The province of Darién presented the highest number of cases per population density in 2015 (4.0) and 2016 (3.9), whereas the province of Veraguas presented the highest number of cases per population density (2.5) in 2017. The number of domestic violence complaints for both sexes was 19 711 and 15 389 in 2016 and 2017, respectively.

## Discussion

Our results indicate that women with the lowest income and educational levels (low and very low) had a higher likelihood of past-year violence (both intimate and non-intimate) and permissive attitudes towards violence, independent of other factors. Moreover, indigenous and Afro-Panamanian ethnicities were associated with physical/sexual IPV. Furthermore, indigenous ethnicity and younger age (15–19 y) were associated with permissive attitudes towards violence.

In the ENVIP study, the lowest monthly income level and divorced/separated status were factors associated with current experiences of violence (either by the intimate partner or not). Gross domestic product per person seems to be a marker of other social processes that often accompany socio-economic development, such as the entry of women into the paid labour force and erosion of the belief in male superiority,^23^ indicating that in addition to focusing on the predominant IPV, action must be taken regarding all forms of violence by creating greater avenues for education, equality and the economic empowerment of women.^24^ Of note, the ENVIP study included only urban areas of the country, therefore results should be interpreted with caution, keeping in mind the findings are more relevant to urban settings. For example, it is conceivable that women with a very high education level are more prone to report violent events. However, the survey provided an opportunity to go beyond IPV and included women >50 y of age, a group often neglected in studies of VAW.^25^ Remarkably, 25.9% of the women exposed to past-year physical/verbal violence were >50 y of age.

Women’s susceptibility to IPV is shown to be greatest in societies where the use of violence is a socially accepted norm.^26^ In line with previous studies, indigenous ethnicity and low educational attainment were associated with a higher likelihood of accepting attitudes towards IPV in the MICS.^27^^,^^28^ Consistent associations were observed for the lowest quintiles of the wealth index (data not shown). Permissive attitudes towards VAW and inequitable gender attitudes have been associated with a greater likelihood of perpetration and social responses to perpetrating physical violence.^29^ In fact, we observed an association between indigenous ethnicity and physical/sexual IPV in the ENASSER. Individuals with permissive attitudes are less likely to report IPV to civil authorities or other family members,^27^ suggesting that men and women in indigenous communities are clear targets for ameliorative efforts taking into account culturally relevant frameworks.^30^ Moreover, Afro-Panamanian ethnicity was associated with physical/sexual IPV. Afro-Panamanians constitute 9.2% of the total population, with higher distributions of Afro-Panamanians in the provinces of Colón and Darién.^31^ Interestingly, both regions had the highest numbers of femicide cases by population density in 2015 and 2016. Likewise, Colón and Darién reported the highest numbers of domestic violence cases, although these data are not reported by sex. Taken together, these results highlight the importance of understanding how social factors contribute to VAW in order to design effective and culturally congruent prevention planning and policies.

Our results indicate that 40% of women experienced controlling behaviours by the intimate partner, a form of violence that is viewed as emotional/psychological abuse. The prevalence of exposure to emotional abuse among women can range from 9% to 70%, and such exposure has a greater impact on women’s mental health than originally thought.^32^ Furthermore, exposure to the controlling behaviour of a partner has been associated with high-risk sexual behaviour.^33^ Future research aimed at disentangling risk indicators associated with this outcome, including the risk factors to assess what leads the partner towards being controlling, is warranted.

Violence during pregnancy has long been neglected; however, negative health effects are particularly amplified during pregnancy.^34^ In Latin America and the Caribbean, prevalence estimates of physical violence during pregnancy vary from 2.5% to 38.7%.^35^ Our prevalence estimate for physical violence was lower than that of a longitudinal study conducted in Brazil (3%),^36^ but our estimates were derived from population-based surveys, and we only assessed physical violence, which limits comparisons. Notably, several studies have reported psychological and sexual violence as most commonly occurring during pregnancy.^37^ Our findings suggest a relationship between low educational attainment and physical violence during pregnancy. Our results are in line with others in which pregnant women with <12 y of education reported a higher prevalence of violence compared with those with >16 y of education.^34^ Future epidemiological/clinical research is needed to implement interventions to prevent and treat the sequelae of violence during pregnancy.

Only a few countries worldwide have specific registries for femicide,^38^ and the age-adjusted femicide rates reported are mostly not standardized. In recent years, the gender equity observatory for Latin America and the Caribbean has been publishing femicide rates at the national level; however, the criteria for femicides within each country might vary.^39^ Brazil reported 4.8 homicides per 100 000 women, whereas Peru reported a femicide rate of <1 per 100 000 women.^40,41^ Panama has recently started to report femicide as an independent cause of death, thus monitoring the geographic disparities among femicide rates is warranted. In recent years the INAMU has expanded its geographic coverage with a multidisciplinary team providing free counselling to women in all provinces and two indigenous territories. Calculating the statistical trends of femicide over time deserves further attention. Another key factor involves the limitations faced by women to report domestic violence.^42^ It should be noted that 50% of global deaths by firearm injuries in 2016 occurred on the American continent, particularly in Latin America, and were driven by homicides, suggesting the need to develop strategies to reduce this form of violence and the arms utilized in them.^43^

Taken together, our results suggest that despite recent advances, prevention programmes to promote non-violent norms regarding masculinity and less passive norms regarding femininity should be advocated.^44^ A complementary and vital approach is for healthcare systems to become more engaged in identifying and responding to violence and participating in prevention efforts.

Several limitations need to be acknowledged. First, temporality cannot be precluded and therefore associations do not infer causation. Second, confounding variables, without overadjusting the models, is difficult to achieve in observational epidemiology, especially with few cases. Although analyses using outcomes with life prevalence experiences have more power, prevention programmes are better informed by looking at risk factors that are amenable to intervention and linked to past-year experiences of violence.^18^ Third, privacy is difficult to maintain in larger and longer surveys, such as the MICS and ENVIP studies, and thus it is likely that prevalence was underestimated. In addition, accepting attitudes towards IPV against women might be underestimated because our outcome measures did not inquire into attitudes about sexual or emotional violence. Fourth, residual or unmeasured confounding needs to be considered when interpreting the results. Nevertheless, to our knowledge, this is the first comprehensive study of VAW in Panama.

## Conclusions

In conclusion, our results indicate that VAW, particularly controlling behaviours by the intimate partner, constitutes a significant problem in Panama. In light of these results, mixed-gender programmes (e.g. balance of power, reduction in stress-producing factors, modification of attitudes) for the prevention of violence in the country, particularly among indigenous communities and Afro-Panamanians, may be of benefit. Future studies aimed at characterizing the risk factors of the violence perpetrator are advocated. To that end, practitioner–researcher collaborations are warranted. From a broader perspective, long-term institutional practices, including the capacity to monitor routine information and evaluate strategies, are strongly recommended.

## Supplementary Material

ihz116_Supplemantary_Files
