# Supplementary material for: Social determinants of violence against women in Panama: results from population-based cross-sectional studies and a femicide registry
Source: Int Health. 2019 Dec 9;14(4):363–72. doi: 10.1093/inthealth/ihz116 (PMC10575601; doi:10.1093/inthealth/ihz116)
Supplement: ihz116_Supplemantary_Files [file ihz116_supplemantary_files.zip › Supplemantary_Figure_2_ihz116.docx]

**Supplemenatry Figure 2. Flowchart summarizing the inclusion and exclusion criteria of the MICS study.**

Answered “I don´t know” (n=84)

*Elegibe women based on the inclusion criteria ([*https://www.contraloria.gob.pa/inec/archivos/MICS_FINAL.pdf-*](https://www.contraloria.gob.pa/inec/archivos/MICS_FINAL.pdf-)*)*
