# Supplementary material for: Social determinants of violence against women in Panama: results from population-based cross-sectional studies and a femicide registry
Source: Int Health. 2019 Dec 9;14(4):363–72. doi: 10.1093/inthealth/ihz116 (PMC10575601; doi:10.1093/inthealth/ihz116)
Supplement: ihz116_Supplemantary_Files [file ihz116_supplemantary_files.zip › Supplemantary_Figure_3_ihz116.docx]

**Supplemenatry Figure 3. Flowchart summarizing the inclusion and exclusion criteria of the ENASSER study.**

*lifetime physical violence during pregnancy, ^&^ as llegally defined.
