# Supplementary material for: Social determinants of violence against women in Panama: results from population-based cross-sectional studies and a femicide registry
Source: Int Health. 2019 Dec 9;14(4):363–72. doi: 10.1093/inthealth/ihz116 (PMC10575601; doi:10.1093/inthealth/ihz116)
Supplement: ihz116_Supplemantary_Files [file ihz116_supplemantary_files.zip › Supplemantary_Table_1_ihz116.docx]

**Supplementary Table 1. Association between SDH and violence against women in the ENASSER study. Odds-ratios (95% confidence interval).**

|  | Controlling attitututes by intimate partner | | Non-partner sexual Violence | |
| --- | --- | --- | --- | --- |
|  | OR 95% CI  (crude) | OR 95% CI  (adjusted)*  **N= 863,103** | OR 95% CI  (crude) | OR 95% CI  (adjusted)*  **N= 1,126,990** |
| Age categories  34-49  25-33  20-24  15-19 | 1  1.2 (0.72.6)  0.9 (0.4-1.9)  1.2 (0.6-2.6) | 1  1.2 (0.7-2.0)  0.9 (0.4-1.7)  1.1 (0.5-2.3) | 1  1.2 (0.5-2.4)  1.0 (0.3-3.7)  0.9 (0.4-2.3) | 1  1.2 (0.5-2.6)  1.1 (0.4-3.2)  1.3 (0.3-4.5) |
| Provinces  Panama  Other Provinces  Bocas and Darien  Indigenous areas | 1  1.3 (0.8-1.9)  1.5 (0.9-2.4)  1.1 (0.7-1.8) | 1  1.2 (0.8-1.9)  1.5 (0.9-2.2)  1.0 (0.5-2.0) | 1  1.5 (0.8-2.9)  2.2 (1.1-4.3)  0.9 (0.4-2.1) | 1  1.6 (0.8-3.1)  1.3 (0.4-3.6)  0.5 (0.1-2.4) |
| Marital status  Married/united  Separated/divorces  Widowed  Single | 1  0.9 (0.5-1.7)  0.9 (0.2-4.0)  - | 1  0.9 (0.5-1.7)  0.8 (0.2-3.8)  - | 1  0.8 (0.4-1.8)  -  0.70 (0.3-1.8) | 1  0.8 (0.4-1.8)  -  0.7 (0.2-2.1) |
| Ethnicity  Other  Indigenous  Afro-panamenian | 1  1.1 (0.7-1.7)  1.2 (0.6-2.3) | 1  0.8 (0.5-1.4)  1.2 (0.6-2.3) | 1  2.0 (0.9-4.8)  0.7 (0.3-1.7) | 1  3.4 (0.9-13.6)  0.9 (0.4-2.5) |
| Educational level  High(reference)  Medium  Low  Very low | 1  1.3 (0.7-2.7)  1.5 (0.8-3.0)  1.2 (0.5-2.8) | 1  1.2 (0.6-2.4)  1.3 (0.7-2.7)  1.1 (0.5-2.6) | 1  0.7 (0.3-1.6)  1.7 (0.7-4.3)  0.9 (0.3-3.1) | 1  0.7 (0.3-1.6)  1.5(0.4-5.3)  0.7 (0.1-3.9) |
| Wealth index  Q5  Q4  Q3  Q2  Q1 | 1  1.4 (0.7-2.8)  1.3 (0.7-2.4)  1.5 (0.8-2.7)  1.5(0.8-2.7) | 1  1.3(0.7-2.2)  1.0 (0.7-1.6)  1.3 (0.8-2.1)  1.5 (0.8-2.6) | 1  0.9 (0.4-2.1)  1.5 (0.7-3.4)  1.6 (0.7-3.6)  0.68 (0.3-1.5) | 1  0.8 (0.4-1.9)  0.9 (0.4-2.1)  0.8 (0.3-2.5)  0.3 (0.09-1.1) |
